# Evaluation of neoadjuvant immunotherapy plus chemotherapy in Chinese surgically resectable gastric cancer: a pilot study by meta-analysis

**DOI:** 10.3389/fimmu.2023.1193614

**Published:** 2023-06-23

**Authors:** Hao Xu, Tengyun Li, Guoyi Shao, Weizhi Wang, Zhongyuan He, Jianghao Xu, Yawei Qian, Hongda Liu, Han Ge, Linjun Wang, Diancai Zhang, Li Yang, Fengyuan Li, Zekuan Xu

**Affiliations:** ^1^ Department of General Surgery, the First Affiliated Hospital of Nanjing Medical University, Nanjing, China; ^2^ Jiangsu Key Lab of Cancer Biomarkers, Prevention and Treatment, Jiangsu Collaborative Innovation Center for Cancer Personalized Medical University, Nanjing, China; ^3^ Department of General Surgery, Jiangyin People’s Hospital Affiliated to Nantong University, Wuxi, China

**Keywords:** resectable advanced gastric cancer, neoadjuvant immunochemotherapy, perioperative immunotherapy, neoadjuant chemotherapy, meta – analysis

## Abstract

**Background:**

Recently, the use of immunochemotherapy in the treatment of advanced gastric cancer (GC) has been increasing and programmed cell death protein 1 (PD-1) inhibitors combined with chemotherapy has become the first-line treatment for advanced GC. However, few studies with small sample sizes have examined this treatment regimen to assess its effectiveness and safety in the neoadjuvant treatment phase of resectable local advanced GC.

**Materials and methods:**

Herein, we systematically searched PubMed, Cochrane CENTRAL, and Web of Science for clinical trials on neoadjuvant immunochemotherapy (nICT) in advanced GC. The primary outcomes were effectiveness [evaluated by major pathological response (MPR) and pathological complete response (pCR)] and safety [assessed by grade 3–4 treatment-related adverse events (TRAEs) and postoperative complications]. A meta-analysis of non-comparative binary results was performed to aggregate the primary outcomes. Direct comparative analysis was used to compare pooled results of neoadjuvant chemotherapy (nCT) with nICT. The outcomes emerged as risk ratios (RR).

**Results:**

Five articles with 206 patients were included, and all of them were from the Chinese population. The pooled pCR and MPR rates were 26.5% (95% CI: 21.3%–33.3%) and 49.0% (95% CI: 42.3%–55.9%), while grade 3–4 TRAEs and post-operative complication rates were 20.0% (95% CI: 9.1%–39.8%) and 30.1% (95% CI: 23.1%–37.9%), respectively. Direct comparison showed that with the exception of grade 3–4 TRAEs and postoperative complications, all outcomes including pCR, MPR, and R0 resection rate favoured nICT to nCT.

**Conclusion:**

nICT is a promising strategy for use as an advisable neoadjuvant treatment for patients with advanced GC in Chinese population. However, more phase III randomized controlled trials (RCTs) will be required to further consolidate the efficacy and safety of this regimen.

## Introduction

In China, gastric cancer (GC) is the fourth most common cancer and third leading cause of cancer-related deaths (1). It is estimated that if gastric cancer risk cannot be effectively controlled, the global burden of gastric cancer is predicted to increase to 1.8 million new cases and 1.3 million deaths by 2040 (2).To date, great progress has been made in understanding the pathogenesis of GC, and surgery remains the backbone of curative treatment (3). Although D2 radical surgery is beneficial, the 5-year survival rate of patients with GC remains below 50% (4). To improve the prognosis of patients with advanced GC, several clinical studies have confirmed that neoadjuvant therapy in locally advanced GC can downstage the tumour, increase the R0 resection rate, and reduce the risk of postoperative recurrence, thereby improving patient outcomes compared with surgery alone (5, 6). Moreover, with ongoing developments in medicine, immunotherapy has started gaining approval in clinical settings, thus changing the landscape of tumour treatment with satisfactory results being observed in the treatment of melanoma and non-small cell lung cancer (7, 8). Immunotherapy has also shown promising results in the treatment of GC. For instance, the Checkmate 649 and Orient-16 studies confirmed that chemotherapy combined with PD-1 inhibitors has significant improvement in overall survival (OS) (HR 0·71;98·4% CI 0·59–0·86; p<0·0001 and HR 0.660; 95% CI 0.505–0.864; P=0.0023, respectively) and progression-free survival (PFS) (HR 0·68; 98% CI 0·56–0·81; p<0·0001 and HR 0.628; 95% CI 0.489–0.805; P=0.0002, respectively) versus chemotherapy alone in patients with a programmed cell death 1 ligand 1(PD-L1) combined positive score (CPS)>5 (9, 10). KEYNOTE-012 and -059 trials confirmed the efficacy of immune checkpoint inhibitors (ICIs) in patients with metastatic GC (11, 12). KEYNOTE-012 reported that overall response was achieved in 8 (22%) of 36 patients while 17 (53%) of 32 patients developed tumour lesion regression. KEYNOTE-059 presented an objective response rate of 15.5% (95% CI 10.1%–22.4%; 23 of 148 patients) in patients with PD-L1-positive tumours. Consequently, immunotherapy is now generally accepted globally as the first-line treatment for advanced GC. However, whether immunotherapy has benefit in the early stages of GC treatment, such as in the neoadjuvant phase, is a current research focus. Furthermore, immunotherapy combined with chemotherapy has been used clinically in GC as neoadjuvant therapy (e.g., in the NCT04354662, NCT04119622, and NCT04694183 trials), while large-scale clinical trials are yet be conducted to assess its efficacy and safety. Hence, this systematic review and meta-analysis of eligible data was performed to assess the efficacy and safety of neoadjuvant immunochemotherapy (nICT) by pathological complete response (pCR), major pathological response (MPR), R0 surgical resection (clinical and complete microscopic resection of the tumour) rate, grade 3–4 treatment-related adverse events (TRAEs), and postoperative complications, in an attempt to provide a more reliable basis for exploring novel therapeutic strategies for GC.

## Materials and methods

### Data sources and search strategy

In current study we followed the Preferred Reporting Items for Systematic Reviews and Meta analyses (PRISMA) and Reporting of Surrogate Endpoint Evaluation using Meta analyses (ReSEEM) guidelines (13, 14). We systematically searched PubMed, Medline, Web of Science and Cochrane Library electronic databases to 1 February 2023 for all clinical trials that tested nICT in advanced GC. The detailed search strategy and inclusion criteria are exhibited in online **supplemental materials**.

### Data extraction

The following variables were extracted from all the included clinical trials, if available: pCR, MPR, R0 surgical resection rate; grade 3–4 TRAEs and incidence of postoperative complications. Other details such as the immune checkpoint inhibitor (ICI) regimen and sample size are also shown in the information sheet.

### Statistical analysis

Data from the individual included studies were entered into a spread sheet for further analysis. Review Manage (RevMan) software version 5.4 was used to perform the statistical analysis. A meta-analysis of the non-comparative binary results was performed based on the most of the involved studies, which were one-arm clinical trials. For evaluating neoadjuvant therapy effectiveness and safety, the aggregated odds ratio (OR) and 95% confidence interval (CI) were transformed into occurrence rates (synthesis of detailed data in the **supplementary information**). *P*< 0.05 for Q test or *I* ^2^ > 50% for *I* ^2^ test was deemed to indicate significant heterogeneity in the literature, random effects model was adopted; otherwise, a fixed effects model was used (15, 16). The level of significance for all results was set at *P* < 0.05. Funnel plots were performed to evaluate possible publication bias (online **Supplemental Figure 2**).

### Risk of bias assessment

Since studies on neoadjuvant immunochemotherapy were mostly non-randomized single-arm clinical trial without comparison groups. Methodological Index for Nonrandomized Studies was used to assess the risk of bias in eligible studies (17).

## Results

### Eligible studies

Five studies (18–22), with a total of 206 enrolled patients were included (**Supplemental Figure 1**). Details of the incorporated studies are shown in **Table 1** and **Supplemental Table 1**. The literature quality of the included studies is summarised in **Supplemental Table 2**.

**Table 1:** Details of extracted data included in the study.

First author	Sample size	Surgery completed	R0	pCR	mPR	III-IVTRAES	Complications
Guo,H.H.	30	30	30	10	19	–	11
Tang,X.H.	75	75	74	21	34	–	22
Lin.J.L.	33	33	32	8	13	–	8
Yu,Y.P.	32	30	30	8	17	4	–
Jiang,H.P.	36	36	35	7	17	10	–

### Evaluation of effectiveness outcomes

**Figure 1:**
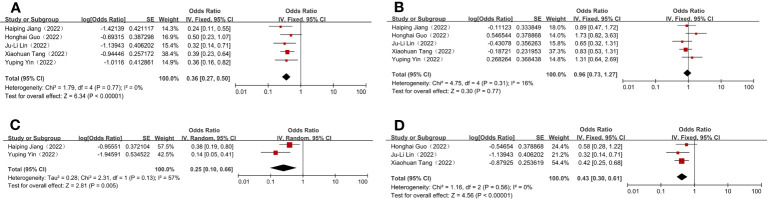
Efficacy and safety evaluation of neoadjuvant immunotherapy plus chemotherapy for locally advanced and resectable gastric cancer. **(A)** Pathological complete response (pCR); **(B)** Major pathological response (MPR); **(C)** Grade 3–4 treatment-related adverse events (TRAEs); **(D)** Surgical complication.

To assess the efficacy of nICT, both the pCR and MPR rates were used. In included studies, the pCR rates ranged from 19.4%–33.3%. The pooled pCR rate was 26.5% (95% CI: 21.8%–33.3%) (**Figure 1A**). In addition, the MPR rates ranged from 39.4%–63.4%, with an aggregated MPR rate of 49.0% (95% CI: 42.3%–55.9%) (**Figure 1B**).

### Evaluation of safety outcomes

The incidence of grade 3–4 TRAEs was recorded as a measure of the safety of nICT, in line with the National Cancer Institute’s Common Terminology Criteria for Adverse Events (NCICT­CAE16; version 4.0). The combined incidence of grade 3–4 TRAEs was 20.0% (95% CI: 9.1%–39.8%) (**Figure 1C**). Three studies reported the precise number of postoperative complications, with the incidences ranging from 24.2%–36.7%, and having a combined incidence of 30.1% (95% CI: 23.1%–37.9%) (**Figure 1D**). Additionally, the R0 resection rates were 100% in most studies; therefore, it was complex to normalise and analyse the extracted data. The incidence of these outcomes is shown in **Table 2**.

**Table 2:** The pairwise comparisons efficacy and safety for neoadjuvant immunotherapy combined with chemotherapy (nICT) and neoadjuvant chemotherapy(nCT).

Pooled iteam	No. of patients						RR(95%CI)	p
	nICT			nCT				
	event	total	rate	event	total	rate		
Efficacy								
pCR	54	204	26.5%	95	1654	5.7%	4.61(3.41,6.23)	<0.00001
MPR	100	204	49.2%	137	584	23.5%	2.09(1.71,2.56)	<0.00001
R0	201	204	98.5%	2109	2456	85.9%	1.15(1.12,1.17)	<0.00001
Safety								
Grade3-4 TRAEs	14	68	20.6%	90	350	25.7%	0.80(0.49,1.32)	0.38
Complications	41	138	29.7%	129	472	27.3%	1.09(0.81,1.46)	0.58

### Direct comparative analysis of neoadjuvant treatments

As most phase II clinical trials were nICT trials in GC, the majority of them were one-arm studies. Owing to this, a network meta-analysis could not be used to compare the efficacy and safety of nICT with that of reported nCT. Thus, based on the existing nCT-included studies (**Supplemental Table 3**), pairwise comparisons were conducted on the incidences of pCR, MPR, R0 resection, as well as grade 3–4 TRAEs and surgical complications associated with nICT and nCT to identify which neoadjuvant therapy regimen was more conducive for patients with GC. The combined outcomes suggested that there were significant differences between nICT and nCT in terms of pCR, MPR, and R0 resection rate, respectively, [(RR = 4.61; 95% CI: 3.41–6.23; p < 0.01), (RR =2.09; 95% CI: 1.71–2.56; p < 0.01), (RR =1.15; 95% CI: 1.12–1.17; p < 0.01)] (**Table 2**). Nevertheless, based on pooled outcomes, grade 3–4 TRAEs and surgical complications did not differ significantly between nICT and nCT, respectively, [(RR = 0.80; 95% CI: 0.49–1.32; p >0.05), (RR =1.09; 95% CI: 0.81–1.46; p >0.05)] (**Table 2**). Taken together, the above results demonstrate that nICT and nCT had comparable rates of grade 3–4 TRAEs and surgical complications, while nICT had higher rates of pCR, MPR, and R0 resection. Therefore, nICT has the potential to be a recommended neoadjuvant treatment for patients with GC.

## Discussion

In the last several years, there has been rapid development of immunotherapy for patients with GC. In 2016, the KEYNOTE-012 trial was the first to demonstrate the potential of GC immunotherapy and lay the foundation for future clinical applications or studies of immunotherapy for GC (12). As third-line therapy, the ATTRACTION-2 study reported superior anti-tumour activity of nivolumab in patients with advanced GC/esophagogastric junction cancer (EGJC) previously treated with chemotherapy (23, 24). In addition, this therapeutic regimen significantly prolonged the survival of patients. KEYNOTE-061 investigated the efficacy of pembrolizumab versus paclitaxel as second-line treatment in patients with PD-1 positive GC/EGJC; nonetheless, the results showed no significant OS improvement with pembrolizumab over paclitaxel (25). Interestingly, a 2020 retrospective study reported by the American Society of Clinical Oncology (ASCO) demonstrated that pembrolizumab significantly prolonged OS and PFS in patients with a high tumour mutation burden (TMB-H) (TMB≥10 mut/MB) in the KEYNOTE-061 cohort (26). Moreover, some studies have found that pembrolizumab could benefit patients with advanced GC with microsatellite instability-high (MSI-H) cancer cells (27, 28). These findings suggest that identifying biomarkers to accurately screen the population with GC that is suitable for immunotherapy is a significant direction in the current research of this disease. The KEYNOTE-062 study was the first multicentre randomised controlled phase 3 clinical trial to evaluate the first-line treatment efficacy of pembrolizumab in patients with GC/EGJC. However, compared to chemotherapy alone, the combination of pembrolizumab and chemotherapy did not result in superior OS and PFS (29). Encouragingly, CheckMate-649, ATTRACTION-04, KEYNOTE-659, and ORIENT-16 reported that first-line treatment with PD-1 inhibitors in combination with chemotherapy could benefit patients with advanced GC (9, 10, 30, 31).

The above reports have prompted several researchers to apply immunotherapy in the neoadjuvant treatment strategy of GC. Furthermore, the curative effect of nICT was preliminarily demonstrated and immunochemotherapy showed great potential (18–22, 32). However, to date, the efficiency and safety of nICT in locally advanced GC have not yet been systematically assessed. Simultaneously, a large number of randomised controlled trials evaluating the clinical efficacy and safety of nICT in GC are lacking. Therefore, this study conducted a quantitative summary of reported studies to provide initial evidence and guidance for use in clinical decision-making during the neoadjuvant treatment of GC. To the best of our knowledge, the present meta-analysis of clinical trials on nICT for resectable advanced GC is the first in its field.

In 1982, Frei et al. first proposed the concept of neoadjuvant chemotherapy(nCT), which refers to systemic chemotherapy given before local treatment (surgery or radiotherapy) of malignant tumors, also known as initial chemotherapy, to show that it is different from postoperative adjuvant chemotherapy (33). Its main purpose is to reduce the volume of tumor lesions in patients or eliminate metastatic cancer cells in advance, which helps to improve the state before surgery and create favorable conditions for subsequent surgery (34). Immunotherapy mainly includes programmed death receptor 1/programmed cell death ligand 1 (PD-1/PD-L1) and cytotoxic T lymphocyte associated antigen-4 (CTLA-4) inhibitors (35, 36). In recent years, immunotherapy has been gradually applied in the treatment of tumors and it has shown unprecedented efficacy in several tumors (7, 8). This has prompted people to combine traditional neoadjuvant chemotherapy with immunotherapy to treat some advanced tumors to form a new neoadjuvant treatment: neoadjuvant immunochemotherapy(nICT) (37).

In this study, the combined rates of pCR, MPR, and R0 for nICT were 26.5%, 49.0%, and 98.5%, respectively, demonstrating the favourable outcome of this therapy in patients with GC. Regarding nCT, pCR, MPR, and R0 rates were 5.7%, 23.5%, and 85.9%, respectively. These outcomes indicated that nICT was superior to nCT, with statistically significant differences being observed (P<0.00001 for all) (**Table 2**). The incidence of grade 3–4 TRAEs and post-operative complications was 20.6% and 29.7% in nICT, and 25.7% and 27.3% in nCT, respectively, with no statistical differences observed (P=0.38, P=0.58, respectively) (**Table 2**). Fortunately, only a few fatal postoperative complications were reported in the included studies, and only one patient died as a result of hemophagocytic syndrome and renal insufficiency (22). In addition, a study of neoadjuvant nivolumab and ipilimumab for resectable GC reported pCR and MPR rates of 58.6% and 72.4%, respectively, indicating that patients obtained a better pathological response, making it easier to achieve a satisfactory prognosis (32). There may be two reasons for the high pCR and MPR rates observed in this aforementioned study. First, this clinical trial used a combination of PD-1 and cytotoxic T lymphocyte-associated antigen-4 (CTLA-4) inhibitors to treat patients with advanced GC. In 2018, the CheckMate-032 study showed that nivolumab alone or nivolumab combined with ipilimumab had high anti-tumour activity and prolonged OS in patients with metastatic esophagogastric cancer (38). Second, the study included patients with deficient mismatch repair/microsatellite instability-high (dMMR)/MSI-H cancer cells, indicating that these patients may have had better responsiveness to immunotherapy (39). Besides, a study on the efficacy of neoadjuvant nivolumab monotherapy for resectable GC showed that the pCR and MPR rates were 3.23% and 16.1%, which were lower than those associated with nICT (40). Further, a previous study demonstrated that nCT enhances the expression of multiple checkpoint molecules and the infiltration of CD4+ and CD8+ immune cells in GC, and the molecular change levels of checkpoints are positively correlated with each other (41). Therefore, ICIs combined with chemotherapy may be more effective than ICIs alone in neoadjuvant treatment of advanced GC. In summary, the above outcomes showed the acceptable efficacy and safety of neoadjuvant immunochemotherapy. Furthermore, it is believed that clinical studies, such as ATTRACTION-05 (42) and KEYNOTE-585 (43) trials, which are currently underway, will provide more evidence on the clinical application of nICT.

There are several limitations to this study. First, in light of the fact that some studies have not reached their endpoints, some survival indicators (such as PFS and OS) could not be investigated. Second, although an extensive literature search was performed, a small number of studies have been included, with inadequate sample sizes and most of them being single-arm studies. Our study also has the following limitations:(I) We conducted a direct pairwise comparison between the nICT and nCT groups and could not fully consider the baseline characteristics between the two sets. (II) The lack of randomised controlled trials (RCTs) may have led to instability and deviations in the study findings. (III) Subgroup analysis of different PD-1 inhibitors was not conducted to evaluate the best immunochemotherapy regimen for clinical application. Furthermore, the patients in this study were all from the Chinese population. The above findings are limited to evaluating the efficacy of neoadjuvant immunochemotherapy for advanced GC in China, which may be difficult to generalize to the whole population. At the same time, we also look forward to more clinical trials of neoadjuvant immunochemotherapy in the treatment of advanced gastric cancer at home and abroad in the future, so as to evaluate its efficacy and safety more comprehensively.

In conclusion, this systematic review and meta-analysis of five non-randomised clinical studies indicated promising effectiveness and safety of nICT in patients with resectable advanced GC in China, providing preliminary clinical evidence for the widespread use of this therapeutic strategy. The results of these studies provide confidence for future research, and RCTs with long-term follow-up are needed to comprehensively evaluate the merits of the nICT for patients with resectable gastric cancer, providing larger sample sizes and complete data to validate the findings of this study.

## Data availability statement

The original contributions presented in the study are included in the article/**Supplementary Material**. Further inquiries can be directed to the corresponding author.

## Author contributions

HX, TL, GS: methodology, data curation, software, writing-original draft. WW, ZH, JX, YQ, HL: conceptualization, investigation, roles/writing—original draft, writing—review and editing. HG, LW, DZ: resources, supervision,validation. LY, FL, ZX: funding acquisition, supervision, writing—review and editing. All authors contributed to the article and approved the submitted version.
