# Supplementary material for: Evaluation of neoadjuvant immunotherapy plus chemotherapy in Chinese surgically resectable gastric cancer: a pilot study by meta-analysis
Source: Front Immunol. 2023 Jun 23;14:1193614. doi: 10.3389/fimmu.2023.1193614 (PMC10326549; doi:10.3389/fimmu.2023.1193614)
Supplement: Supplementary file 1 [file DataSheet_1.pdf]

Online Supplemental Table3 Main characteristics of included the studies of neoadjuvant chemotherapy.

First author	PMID	Study type	Study phase	Recruitment criteria	Regimen	pCR/Size	MPR/Size	R0/Size	TRAEs (grade3-4)/Size	postoperative complications/Size
T. Aoyama	28486692	Cohort study	II	IIA-IIIC	Cisplatin/S-1 (CS) and docetaxel/cisplatin/S-1 (DCS)	11/127	22/127	102/132	-	-
B. Brenner	16788003	Cohort study	II	IIA-IIIC	Cisplatin, fluorouracil	-	-	32/38	-	10/34
C.Schuhmacher	21060024	Cohort study	II	IIA-IV	Cisplatin, d-L-folinic acid, fluorouracil	-	-	59/70	-	19/70
B.Kumar Sah	33257672	Cohort study	II	IIB-IVA	Docetaxel, oxaliplatin, fluorouracil, leucovorin/ S-1 plus oxaliplatin	1/74	19/74	51/55	14/55	16/55
T Yoshikawa	19644974	Cohort study	II	IA-IIIC	Irinotecan, cisplatin	1/47	-	36/46	-	-
U Fink	7552009	Cohort study	II	IIIA-IV	etoposide, doxorubicin ,cisplatin	-	-	24/27	-	-
A Tsuburaya	24668391	Cohort study	II	NR	S-1 and cisplatin	1/49	--	43/49	-	-
T Yoshikawa	27244537	Cohort study	II	III	S-1 plus cisplatin (SC) and paclitaxel plus cisplatin (PC)	-	-	62/77	-	-
S Watson	30529902	Cohort study	II	II-III	Oxaliplatin, 5-Fluorouracil and Nab-paclitaxel	8/49	19/49	42/44	-	14/44
X Wang	27172250	Cohort study	II	IB-III	mFOLFOX6	2/67	-	67/72	-	-
Y Iwasaki	33200303	Cohort study	III	III-IV	S-1plus cisplatin	3/139	-	112/139	-	-
Y Kurokawa	23999869	Cohort study	II	IB-IIIC	S-1plus cisplatin	2/100	-	73/100	-	-
M Terashima	30827001	Cohort study	III	III-IV	S-1 plus cisplatin	-	-	112/139	-	-
K Hosoda	30284080	Cohort study	II	IIA-IIIC	docetaxel, cisplatin, and S-1	3/40	-	36/40	-	-
M Iwatsuki	34379229	Cohort study	II	II-III	S-1 and oxaliplatin	4/42	-	41/42	-	-
T Kinoshita	19390930	Cohort study	II	NR	S-1- 5-fluorouracil	-	-	-	3/55	11/46
C Schulz	25530271	Cohort study	II	IIA-IIIC	oxaliplatin ,leucovorin, 5fluorouracil ,docetaxel	10/50	20/50	43/50	-	-
J Matsuyama	35380725	Cohort study	II	IIB-IIIC	capecitabine plus oxaliplatin	1/37	3/37	29/33	-	-

R Biffi	20143466	Cohort study	II	IB-III	Docetaxel,cisplatin, fluorouracil	4/32	-	29/32	-	9/32
Y-J Yu	25561810	Cohort study	II	III-IV	Docetaxel,cisplatin, fluorouracil	1/51	-	31/51	-	-
Z-F Wu	26620627	Cohort study	NR	NR	Epirubicin, oxaliplatin, capecitabine	4/178	-	178/178	-	-
S-S Chen	23879173	Cohort study	II	IB-IIIC	FOLFOX6	-	-	15/19	-	-
Q Zhao	32583567	Cohort study	III	IIA-IIIC	S-1 plus oxaliplatin and capecitabine plus oxaliplatin	-	-	394/459	-	-
P M Schneider	29743108	Cohort study	II	II-III	Oxaliplatin, docetaxel, 5-FU	2/30	11/30	25/30	-	-
E Oki	24604583	Cohort study	II	IIIA-IIIB	docetaxel and S-1	2/47	-	-	-	-
T Yoshikawa	23838904	Cohort study	II	IIA-IIIC	S-1/cisplatin and paclitaxel/cisplatin	4/83	-	62/83	-	-
M Menges	12836016	Cohort study	II	III-IV	cisplatin, 5-fluorouracil and folinic acid	-	-	13/20	-	-
A Serizawa	34302539	Cohort study	II	II-III	S-1 plus oxaliplatin	2/30	-	28/30	-	-
K Sasaki	28707042	Cohort study	II	IIB-IIIC	docetaxel, cisplatin, and S-1	5/30	-	28/30	-	-
R Berenato	28881354	Cohort study	II	IIA-IIIC	Capecitabine, Oxaliplatin, Irinotecan	2/40	-	33/40	-	-
C Barone	15459495	Cohort study	II	IIA-IIIC	epidoxorubicin, etoposide and cisplatin	-	-	20/24	-	5/24
T Kosaka	24253176	Cohort study	II	III	S-1 and docetaxel	1/14	-	14/14	-	-
A Tsuburaya	23463482	Cohort study	II	III-IV	paclitaxel and cisplatin	1/50	5/50	33/43	20/52	-
I Park	23921575	Cohort study	II	II-IV	docetaxel, oxaliplatin, and S-1	6/41	-	40/41	23/41	-
M Hirakawa	23338051	Cohort study	II	II-IV	docetaxel, cisplatin, and S-1	-	-	39/42	-	-
Y-K Kang	20811894	Cohort study	II	NR	docetaxel, capecitabine, and cisplatin	4/40	-	-	-	-
J-L Lin	35116023	Cohort study	NR	II-III	S-1 + oxaliplatin and S-1 + nab-paclitaxel	10/167	38/167	163/167	-	45/167

pCR =pathological complete response; MPR = major pathological response; TRAEs = treatment-related adverse events; NR = not reported.
