# Supplementary figures and images for: Evaluation of neoadjuvant immunotherapy plus chemotherapy in Chinese surgically resectable gastric cancer: a pilot study by meta-analysis
Source: Front Immunol. 2023 Jun 23;14:1193614. doi: 10.3389/fimmu.2023.1193614 (PMC10326549; doi:10.3389/fimmu.2023.1193614)

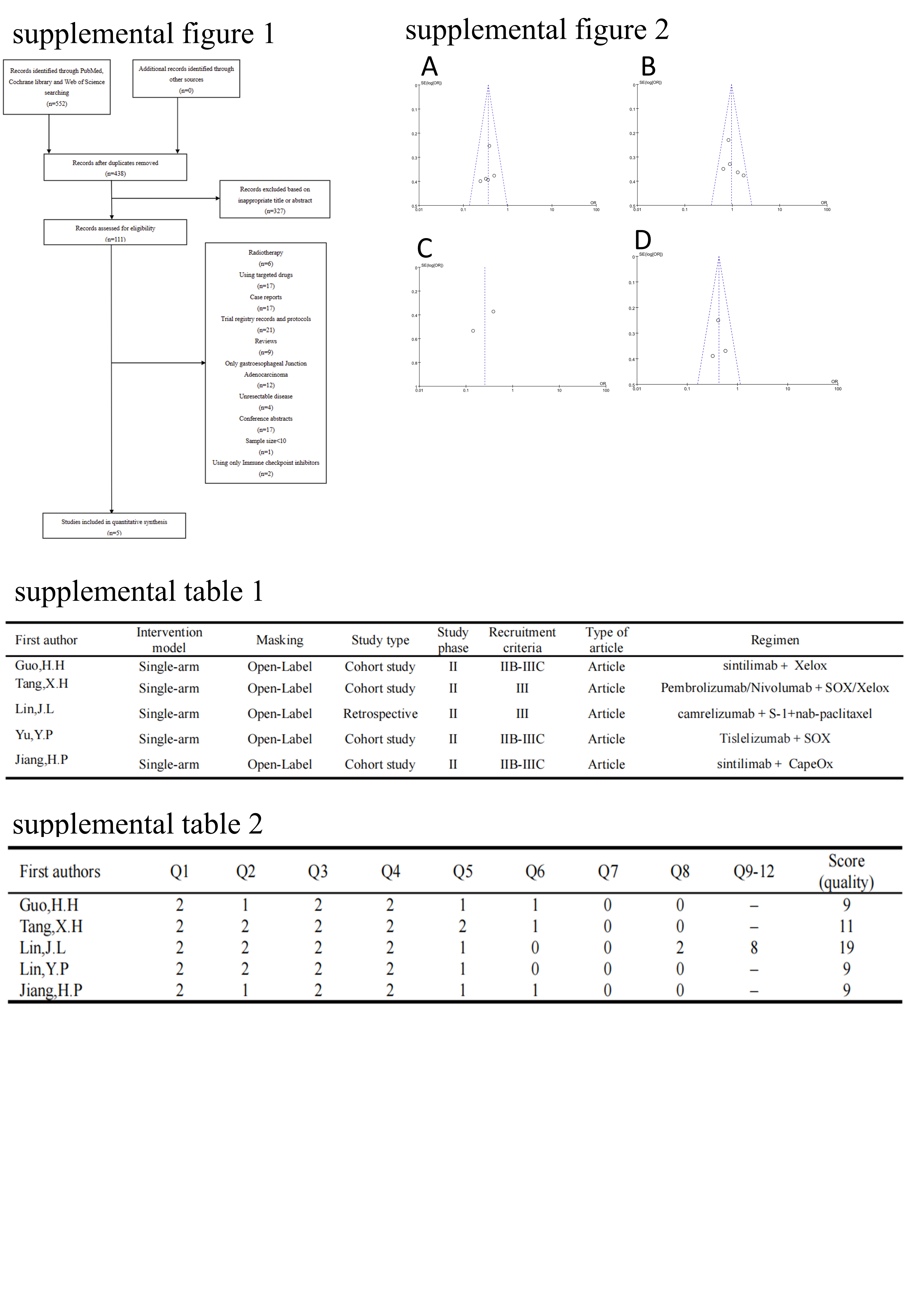

Supplement: Supplementary file 2 [file Image_1.tif]
