# Supplementary material for: Evaluation of neoadjuvant immunotherapy plus chemotherapy in Chinese surgically resectable gastric cancer: a pilot study by meta-analysis
Source: Front Immunol. 2023 Jun 23;14:1193614. doi: 10.3389/fimmu.2023.1193614 (PMC10326549; doi:10.3389/fimmu.2023.1193614)
Supplement: Supplementary file 3 [file Table_1.docx]

Supplemental materials

Contents

1.Search strategy................................................................................................2

2.Data Synthesis.................................................................................................4

3.The inclusion criteria.......................................................................................5

4.Characteristics of included studies..................................................................6

5.Risk of bias in cohort study.............................................................................6

6.Supplemental figure legend……………………………………………….....7

7.Supplemental figure.........................................................................................7

Search strategy

1.Search strategy for Pubmed :

**(((((((((((((((PD-1 checkpoints inhibitors[Title/Abstract]) OR (PD-1 blockade[Title/Abstract])) OR (Nivolumab[Title/Abstract])) OR (pembrolizumab[Title/Abstract])) OR (lambrolizumab[Title/Abstract])) OR (sintilimab[Title/Abstract])) OR (camrelizumab[Title/Abstract])) OR (Cemiplimab[Title/Abstract])) OR (PD-L1 blockade[Title/Abstract])) OR (PD-L1 checkpoints inhabitors[Title/Abstract])) OR (Atezolizumab[Title/Abstract])) OR (durvalumab[Title/Abstract])) OR (avelumab[Title/Abstract])) OR (toripalimab[Title/Abstract])) OR (tislelizumab[Title/Abstract])) AND ((((((((stomach neoplasms[Title/Abstract]) OR (stomach carcinoma[Title/Abstract])) OR (stomach adenocarcinoma[Title/Abstract])) OR (stomach tumor[Title/Abstract])) OR (gastric carcinoma[Title/Abstract])) OR (gastric adenocarcinoma[Title/Abstract])) OR (gastric tumor[Title/Abstract])) OR (gastric cancer[Title/Abstract]))) AND ((((((((((((((neoadjuvant[Title/Abstract]) OR (neoadjuvant therapy[Title/Abstract])) OR (preoperative[Title/Abstract])) OR (preoperation[Title/Abstract])) OR (pre-operation[Title/Abstract])) OR (pre-op[Title/Abstract])) OR (before the operation[Title/Abstract])) OR (before the surgery[Title/Abstract])) OR (before operations[Title/Abstract])) OR (before surgeries[Title/Abstract])) OR (prior to the operation[Title/Abstract])) OR (prior to the surgery[Title/Abstract])) OR (prior to operations[Title/Abstract])) OR (prior to surgeries[Title/Abstract]))**

2.Search strategy for Web of science:

**TS=(immunotherapy OR PD-1 checkpoints inhibitors OR PD-1 blockade OR Nivolumab OR pembrolizumab OR lambrolizumab OR sintilimab OR camrelizumab OR Cemiplimab OR PD-L1 blockade OR PD-L1 checkpoints inhabitors OR Atezolizumab OR durvalumab OR avelumab OR toripalimab OR tislelizumab) AND TS=(stomach neoplasms OR stomach carcinoma OR stomach adenocarcinoma OR stomach tumor OR gastric carcinoma OR gastric adenocarcinoma OR gastric tumor OR gastric cancer) AND TS=(neoadjuvant OR neoadjuvant therapy OR preoperative OR preoperation OR pre-operation OR pre-op OR before the operation OR before the surgery OR before operations OR before surgeries OR prior to the operation OR prior to the surgery OR prior to operations OR prior to surgeries)**

3.Search strategy for Cochrane CENTRAL

**(stomach neoplasms OR stomach carcinoma OR stomach adenocarcinoma OR stomach tumor OR gastric carcinoma OR gastric adenocarcinoma OR gastric tumor OR gastric cancer):ti,ab,kw AND (immunotherapy OR PD-1 checkpoints inhibitors OR PD-1 blockade OR Nivolumab OR pembrolizumab OR lambrolizumab OR sintilimab OR camrelizumab OR Cemiplimab OR PD-L1 blockade OR PD-L1 checkpoints inhabitors OR Atezolizumab OR durvalumab OR avelumab OR toripalimab OR tislelizumab):ti,ab,kw AND (neoadjuvant OR neoadjuvant therapy OR preoperative OR preoperation OR pre-operation OR pre-op OR before the operation OR before the surgery OR before operations OR before surgeries OR prior to the operation OR prior to the surgery OR prior to operations OR prior to surgeries):ti,ab,kw**

**Data Synthesis：**

Pathological complete response (pCR) and major pathologic response (MPR) were used to evaluate the efficacy of neoadjuvant immunotherapy. Safety of neoadjuvant immunotherapy assessed by the incidence of grade 3-4 TRAEs and surgical complications. When the results of the included studies do not accord with the normal distribution, it is necessary to perform Freeman-Tukey double-arcsine transformation for raw incidence rates^1^. The calculation of the effect index (P) and its standard error SE (P) of non-comparative binary outcomes was performed according to the formula: P=ln(odds)=ln(X/(n–X)). SE(P)= SE(ln(odds)) =$\sqrt{1/{X+1/{(n-X)}}}$. The combined odds ratio (OR) and 95% confidence interval (CI) need to be converted into the incidence rate (Pf=OR/(1+OR);95 % CI: LL=LL_OR_/ (1+LL_OR_), UL=UL_OR_/ (1+ UL_OR_)^2^. The incidence of events between any two groups was compared by risk ratio(RR) and its 95% confidence interval (CI). RR and 95% CI > 1 indicates that the incidence of events is lower than the control group, while < 1 indicates that the incidence is higher than the control group.

**The inclusion criteria**

1. prospective phase I, phase II or phase III clinical trials for pathologically diagnosed stage I-III resectable GC;
2. neoadjuvant therapy with ICI and chemotherapy
3. resporting pCR, MPR, R0, grade 3 and 4 TRAEs and incidence of surgical complications as clinical outcomes.

**Supplementary Table 1** Characteristics of included studies.

**Supplemental Table 2** Assessment of the quality of included studies according to MINORS.

Quality of non-comparative studies was determined based on the first eight items; the last four items were only used to assess comparative studies.

Checklist items: 1, a stated aim of the study; 2, inclusion of consecutive patients; 3, prospective collection of data; 4, endpoints appropriate to study aim; 5, unbiased assessment of study endpoint; 6, follow-up period appropriate to the major endpoint; 7, <5% lost to follow-up; 8, adequate control group; 9, contemporary groups; 10, baseline equivalence of groups; 11, prospective calculation of study size; 12, adequate statistical analyses. Items are scored as 0 (not reported); 1 (reported but inadequate); or 2 (reported and adequate). The maximum possible score is 24 points.For non-comparative studies, an overall score > 12 = high; 8–12 = intermediate; < 8 = low. For comparative studies, > 18 = high; 12–18 = intermediate; < 12 = low.

**Supplemental figure legend**

**Supplemental figure 1**

Flow diagram of publication search

**Supplemental figure 2**

**Supplemental figure 2A** Funnel plot of complete pathological response

**Supplemental figure 2B** Funnel plot of major pathological response

**Supplemental figure 2C** Funnel plot of grade 3 to 4 treatment-related adverse events

**Supplemental figure 2D** Funnel plot of surgical complications

**References:**

1. Masarwy R, Kampel L, Horowitz G, et al. Neoadjuvant PD-1/PD-L1 Inhibitors for Resectable Head and Neck Cancer: A Systematic Review and Meta-analysis. JAMA Otolaryngol Head Neck Surg 2021; 147(10):871-878.

2. Chen YH, Du L, Geng XY, Liu GJ. Implement meta-analysis with non-comparative binary data in RevMan software. Chin J Evid Based Med 2014; 14(7):889-896.
